# The role of aedeagus size and shape in failed mating interactions among recently diverged taxa in the *Drosophila mojavensis* species cluster

**DOI:** 10.1186/s12862-014-0255-3

**Published:** 2014-12-10

**Authors:** Maxi Polihronakis Richmond

**Affiliations:** Division of Biological Sciences, University of California, San Diego, 9500 Gilman Dr., La Jolla, CA 92093 USA

**Keywords:** Pseudocopulation, *Drosophila mojavensis*, *Drosophila arizonae*, Mating behavior, Genitalia evolution

## Abstract

**Background:**

Investigating the evolution of species-specific insect genitalia is central to understanding how morphological diversification contributes to reproductive isolation and lineage divergence. While many studies evoke some form of sexual selection to explain genitalia diversity, the basis of selection and the mechanism of heterospecific mate exclusion remains vague. I conducted reciprocal mate pair trials in the *Drosophila mojavensis* species cluster to quantify the frequency of failed insemination attempts, historically referred to as pseudocopulation, between lineages with discrete size and shape differences of the male aedeagus.

**Results:**

In cross-taxon matings aedeagus size had a significant effect on pseudocopulation frequencies, while aedeagus shape and genetic distance did not. The direction of the size difference was an important factor for successful mating. When females were mated to a cross-taxon male with a larger aedeagus than males from her own species, the pair could not establish a successful mating interaction. Females mated to cross-taxon males with a smaller aedeagus than conspecific males were able to establish the mating interaction but had issues disengaging at the end of the interaction.

**Conclusions:**

The results of this study support a role for aedeagus size in the male-female mating interaction, with a secondary role for aedeagus shape. In natural populations, mating failure based on aedeagus size could serve as an important reproductive isolating mechanism resulting in failed insemination attempts after both the male and female show a willingness to mate.

**Electronic supplementary material:**

The online version of this article (doi:10.1186/s12862-014-0255-3) contains supplementary material, which is available to authorized users.

## Background

Much of the intrigue underlying research of animal genitalia is driven by a desire to understand the evolutionary forces responsible for these diverse, complex, and remarkably intricate structures. Because insect genitalia are often species-specific, investigating genitalia diversity can enhance our understanding of the putative role these structures play in reproductive isolation and lineage divergence. However, the structural complexity of genitalia, and the numerous types of mating systems they operate in, has made it difficult to determine the proximate mechanisms involved. While sexual selection is largely agreed upon as a general explanation, results from numerous studies covering a wide diversity of insect groups vary in their support for more specific explanations such as sperm competition, female choice, lock-and-key, or sexual conflict. In a recent review, Simmons [1] reiterated that these explanations are not mutually exclusive and described their roles on a continuum of sexual selection processes.

Insect genitalia comprise a functionally integrated unit with multiple parts that can differ in both size and shape. In general, genital size in insects is negatively allometric within species, and thus does not vary with body size like most morphological traits [2-4]. Shape, on the other hand, can be highly variable within species [5]. There is evidence that genitalia size and shape evolve independently and could thus have different effects on the mating interaction [6-8]. Numerous studies have demonstrated the effect of size and shape variation on mating success, sperm transfer, sperm storage, and paternity (reviewed in [1]).

In the current study, I investigated the role of aedeagus size and shape in failed mating interactions among taxa in the *D. mojavensis* species cluster. Previous experiments of cross-taxon mate trials resulted in several observations where pre-copulatory barriers were overcome, i.e. females accepted courting males, but mating pairs failed to achieve the appropriate copulatory configuration [9]. The resulting “pseudocopulations” were typically very short in duration (2-10 seconds), and no offspring were produced [10]. Because of the potential for failed mating interactions to cause reproductive isolation among taxa in the *D. mojavensis* species cluster, the current study tests how lineage specific aedeagus variation influences the frequency of pseudocopulation.

**Figure 1:**
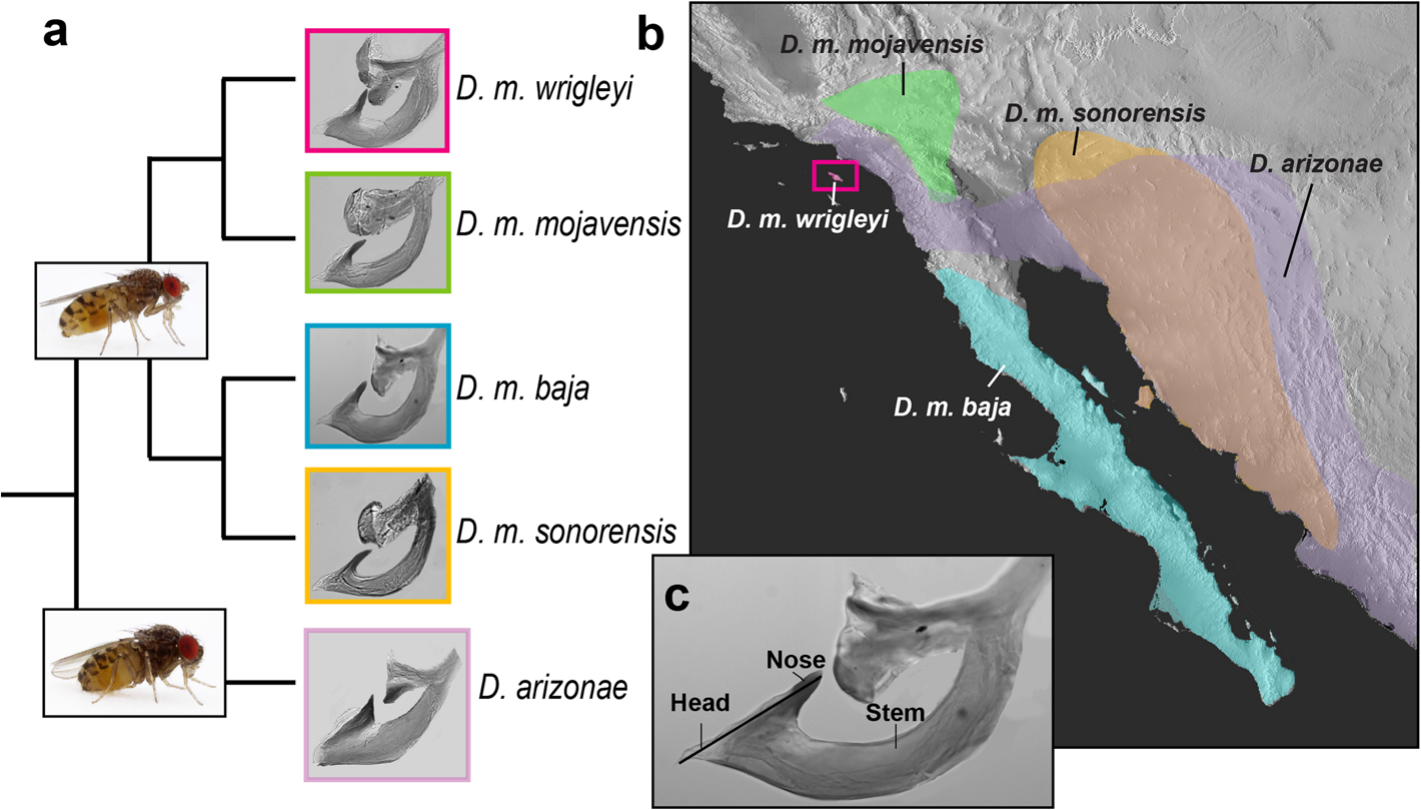
***D. mojavensis*** **species cluster. a)** Phylogenetic relationships (from Machado et al. [11]) with lateral habitus images of adults, and images of the male aedeagus for each group. **b)** Corresponding geographic distribution for each taxon in the phylogeny, and **c)** Image of aedeagus illustrating regions used in the text to describe shape differences: “head” (including measurement line indicating how size was measured), “nose”, and “stem”.

*Drosophila mojavensis* comprises four described subspecies based on phylogenetic and population genetic analyses of geographically isolated populations that specialize on different host cacti [11-13] (Figure 1). Studies quantifying the extent of reproductive isolation among the four *D. mojavensis* subspecies have revealed varying levels and types of reproductive isolation depending on which populations of a particular sex are involved [14-18]. *Drosophila arizonae*, the sister species to *D. mojavensis*, will feed and breed on columnar cactus species but also is a generalist on a wide variety of food-types [19]. The varying degrees of reproductive isolation among the *D. mojavensis* subspecies, and between *D. mojavensis* and *D. arizonae*, have facilitated use of this system as a model to study incipient speciation and the chronology of lineage divergence processes that accompany ecological host shifts. More recently, the *D. mojavensis* species cluster has been used to study the corresponding divergence of morphological characters [20], which revealed discrete variation of the male aedeagus between *D. arizonae* and *D. mojavensis*, and among three of the four *D. mojavensis* subspecies (*D. m. mojavensis*, *D. m, baja*, and *D. m. wrigleyi*).

To test whether aedeagus size and/or shape play a role in the male-female mating interaction and mate recognition in the *D. mojavensis* cluster, I quantified failed genitalic interactions, or pseudocopulation, in reciprocal pairwise mate trials among all *D. mojavensis* subspecies and *D. arizonae*. Pseudocopulation rates were used in conjunction with morphological and genetic data from previous studies [11,20] to test the role of size, shape, and genetic relatedness on mating success. I found that size, but not shape or genetic distance, had a significant effect on the frequency of pseudocopulation. Thus, it appears that aedeagus size in the *D. mojavensis* species cluster plays a role in mate recognition and has the potential to cause reproductive isolation among lineages with sufficient size differences.

## Results

### Pseudocopulation

The sequence of events in a mate trial scored as pseudocopulation was as follows: a female accepted a courting male by spreading her wings and lowering her abdomen, the male mounted the female and tucked the tip of his abdomen downward and anteriorly to insert his aedeagus into the female vagina. When the couple was unable to establish a connection between their reproductive organs the male fell off and this was scored as pseudocopulation (Additional file 1: Video S1). The duration of pseudocopulation ranged between 3 and 29 seconds versus 80 – 270 seconds for normal copulations. Two types of pseudocopulation were scored, those that did not result in a successful mating after three attempts (Type I), and those where a normal mating was preceded by up to three unsuccessful mounting attempts (Type II). Normal and Type II matings yielded progeny; however, no Type I pseudocopulation yielded progeny. In nature, Type I pseudocopulation could thus serve as an important reproductive isolating mechanism that results in failed insemination attempts after both the male and female show a willingness to mate.

**Figure 2:**
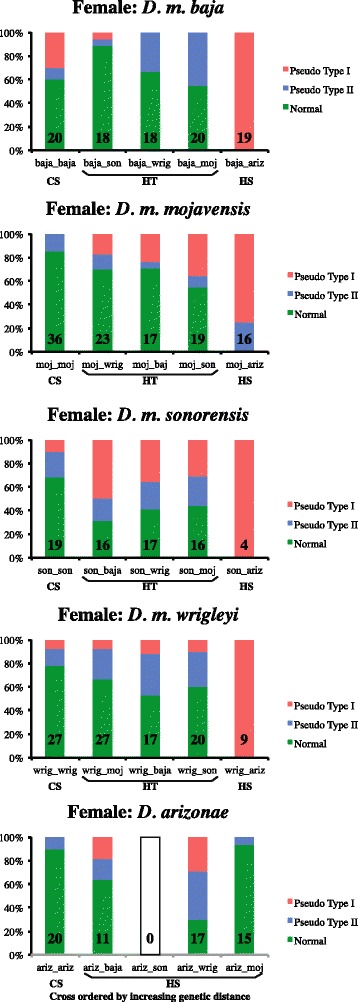
**The frequency of each of the three possible mating outcomes grouped by female taxonomic identity.** CS = conspecific, HT = heterotypic, HS = heterospecific. The number within each bar represents the number of trials where the male courted, the female accepted, and a mating attempt occurred (= denominator in all frequency calculations).

The total number of mate trials, and number of trials where males courted and the female accepted are provided in Additional file 2: Table S2. The latter number was used as the denominator for calculating the frequency of normal copulation, and Type I and Type II pseudocopulation (Figure 2). The frequency of Type I pseudocopulation was low in all conspecific pairings, except in *D. m. baja*, which had a much higher frequency of Type I pseudocopulation (30.0%) than all other taxa (Table 1 and Figure 2). When male *D. m. baja* were paired with other *D. mojavensis* subspecies (heterotypic matings) *and D. arizonae* (heterospecific matings), the frequency of Type I pseusocopulation was lower than in conspecific trials, except for trials with female *D. m. sonorensis*. When female *D. m. baja* were paired with other *D. mojavensis* subspecies the frequency of Type I pseudocopulation was also lower than in conspecific trials, but not for trials with *D. arizonae* in which all mating attempts resulted in Type I pseudocopulation.

**Table 1:** **Percentage of Type I pseudocopulation by cross type**

	**Male**	***D. m. baja***	***D. m. mojavensis***	***D. m. sonorensis***	***D. m. wrigleyi***	***D. arizonae***
**Female**						
***D. m. baja***		30.0	0.0	5.6	0.0	100.0
***D. m. mojavensis***		23.5	0.0	35.0	17.4	75.0
***D. m. sonorensis***		50.0	31.3	10.5	35.3	100.0
***D. m. wrigleyi***		11.8	7.4	10.0	7.4	100.0
***D. arizonae***		18.2	0.0	N/A	29.4	0.0

In heterotypic crosses, the frequency of Type I pseudocopulation was highest between *D. m. sonorensis* females and *D. m. baja* males (50.0%) (Table 1 and Figure 2). While females accepted male courtship 70% of the time, half of the copulation attempts resulted in pseudocopulation. In the remaining trials, 18.8% resulted in Type II pseudocopulation and 31.3% mated successfully on the first try.

The majority of heterospecific copulatory attempts between *D. mojavensis* subspecies females and *D. arizonae* males resulted in Type I pseudocopulation (Table 1 and Figure 2). In the case of *D. m. sonorensis* and *D. m. wrigleyi*, less than half of the pairs even got to this point in the mating sequence (i.e. in *D. m. sonorensis* only four of 20 females accepted courting males, but all resulted in pseudocopulation). On the other hand, in trials between *D. m. baja* females and *D. arizonae* males, females accepted 95% of courting males but all resulted in Type I pseudocopulation. In general, heterospecific mate trials with *D. arizonae* females resulted in lower Type I pseudocopulation frequencies than the reciprocal crosses summarized above. *Drosophila arizonae* females did not accept courtship from *D. m. sonorensis* males precluding an assessment of pseudocopulation in these pairings.

In order to assess lineage specific variation in Type I pseudocopulation frequency, I grouped mate trials by taxon and tested for differences using Fisher’s exact test (Tables 2 and 3). For example, the first row in Table 2 shows the frequency of Type I pseudocopulation in all crosses where the male was *D. m. baja*. When trials were grouped by male identity, there was a significant difference in Type I pseudocopulation frequencies among taxa for conspecific and heterospecific trials, but not for heterotypic trials (Table 2). I also looked at comparisons among mate groups within each taxon. Conspecific, heterotypic, and heterospecific trials with *D. mojavensis* subspecies males did not have significantly different frequencies of Type I pseudocopulation. However, Type I pseudocopulation frequencies between conspecific and heterospecific trials involving *D. arizonae* males were significantly different (Table 2). In sum, when trials were grouped by male taxonomic identity the frequency of Type I pseudocopulation only varied significantly in trials involving male *D. arizonae*.

**Table 2:** **Percentage of Type I pseudocopulation grouped by male taxon (all mate trials involving male** ***D. m. baja*** **, male** ***D. m. mojavensis*** **, etc.), for each mate group (CS = conspecific pairing, HT = heterotypic pairing HS = heterospecific pairing)**

**Species**	**%CS^**	**%HT**	**%HS^**
*D. m. baja*	30.0	28.0	18.2
*D. m. mojavensis*	0.0	11.1	0.0
*D. m. sonorensis*	10.5	17.2	N/A
*D. m. wrigleyi*	7.4	17.2	29.4
D. arizonae*	0.0	-	91.7

* = significant among mating group type (horizontal), ^ = significant among taxa (vertical) using Fisher’s Exact test for multiple pairwise comparisons with Bonferroni correction (α = 0.05). (“-” denotes no HT cross exists for *D. arizonae.*).

**Table 3:** **Percentage of Type I pseudocopulation grouped by female taxon (all mate trials involving female** ***D. m. baja*** **, male** ***D. m. mojavensis*** **, etc.), for each mate group (CS = conspecific pairing, HT = heterotypic pairing HS = heterospecific pairing)**

**Species**	**%CS^**	**%HT^**	**%HS^**
D. m. baja*	30.0	1.8	100.0
D. m. mojavensis*	0.0	25.0	75.0
D. m. sonorensis*	10.5	38.8	100.0
D. m. wrigleyi*	7.4	9.4	100.0
*D. arizonae*	0.0	-	16.3

* = significant among mating group type (horizontal), ^ = significant among taxa (vertical) using Fisher’s Exact test for multiple pairwise comparisons with Bonferroni correction (α = 0.05). (“-” denotes no HT cross exists for *D. arizonae.*).

When trials were grouped by female identity, a different picture emerged. First, the frequency of pseudocopulation was significantly different among taxa within each mate group type (Table 3). For example, the frequency of Type I pseudocopulation in heterotypic trials was significantly different among the *D. mojavensis* subspecies, which was not the case when trials were grouped by male identity. Trials with *D. m. sonorensis* and *D. m. mojavensis* females had the highest frequency of Type I pseudocopulation. In comparisons among mate group type within each taxon, there were significant differences for each of the *D. mojavensis* subspecies; meaning, within each subspecies the frequency of Type I pseudocopulation differed among conspecific, heterotypic, and heterospecific trials. This was not the case when trials were grouped by male identity. In trials with *D. arizonae* females, there was no significant difference between conspecific and heterospecific trials. This is interesting because *D. arizonae* males have a larger aedeagus than *D. mojavensis* subspecies males, and suggests Type I pseudocopulation results when females are attempting to mate with males that have a larger aedeagus than their own males. In sum, these data highlight the importance of male genitalia size from the perspective of the female in Type I pseudocopulation frequencies.

In several matings between *D. arizonae* females and *D. mojavensis* subspecies males, the male and female were unable to disengage their reproductive organs at the end of the copulatory interaction (Additional file 3: Video S2). In some cases, the male position would change so that instead of facing the same direction as the female, he would turn 180° and face away from the female while the reproductive organs were still engaged. This often resulted in the female dragging the male around before being able to separate. Females that experienced disengagement issues were able to lay eggs, but none of these heterospecific crosses yielded progeny. The amount of time males were stuck ranged from five seconds to 12 min. 57 sec. There were no observed instances of *D. arizonae* males getting stuck in copulatory interactions with any *D. mojavensis* subspecies, but few chances occurred (only four instances of Type II pseudocopulation between *D. m. mojavensis* females and *D. arizonae* males, and no normal copulations*).* Disengagement issues were not observed in any conspecific or heterotypic mate interactions.

### Genetic and morphological distances

**Figure 3:**
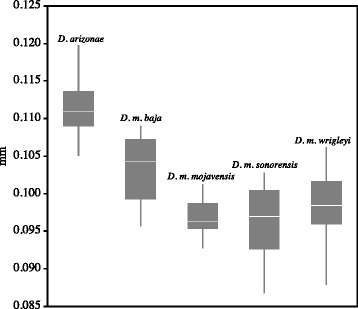
**Box plot of aedeagus size.** Data from Richmond et al. [20]. The white line within each box is the mean, the box is the 25%-75% quartiles, and the lines show the minimum and maximum values.

The species tree topology was the same as the gene tree presented in Machado et al. [11] (Additional file 2: Figure S1). *Drosophila arizonae* was sister to all four *D. mojavensis* subspecies. Within *D. mojavensis*, *D. m. wrigleyi* was sister to *D. m. mojavensis,* and *D. m. sonorensis* was sister to *D. m. baja*. Mahalanobis distances representing morphological differences in aedeagus shape were the greatest between *D. arizonae* and *D. m. baja*, followed by *D. arizonae* and *D. m. sonorensis* (Additional file 2: Table S8). Within *D. mojavensis*, the greatest aedeagus shape differences were between *D. m. wrigleyi* and *D. m. baja*, followed by *D. m. mojavensis* and *D. m. baja*. The aedeagus of *D. arizonae* was bigger than all other *D. mojavensis* subspecies (Figure 3). Within *D. mojavensis*, *D. m. baja* had the largest aedeagus and the greatest size difference was between *D. m. sonorensis* and *D. m. baja* followed by *D. m. wrigleyi* and *D. m. baja*.

### Linear regression

The results of the two multiple linear regression analyses (Type I pseudocopulation as the dependent variable; and Type I plus Type II pseudocopulation as the dependent variable) were similar; thus, only results of the analysis with Type I pseudocopulation are presented here. The adjusted *r*^2^ value (0.834) was significant (*F* = 39.52, p < 0.0001). The coefficients (and associated standard error) for the linear regression were 0.1426 (0.01822), 4.01927 (2.80370), and 7.33298 (10.58423) for size difference, aedeagus shape distance, and genetic distance, respectively. When controlling for genetic and shape differences, size was the only significant variable affecting Type I pseudocopulation rate (*t* = 7.825, p < 0.0001). When controlling for size difference and genetic distance, the effect of shape was not significant (*t* = 1.434, p = 0.1671). When controlling for size and shape, the effect of genetic distance was not significant (*t* = 0.693, p = 0.4964). Due to the potential for non-independence among cross-types as a result of conducting reciprocal crosses, randomization tests were used to verify the significance values of each independent variable in the linear regression. In each of 10,000 replicates, pseudocopulation frequencies were randomized among crosses and regression coefficients were calculated. Significance was determined by comparing the coefficients from the linear regression to the distribution produced from the 10,000 randomized data sets. The coefficients for aedeagus shape difference and genetic distance remained non-significant. The coefficient for aedeagus size was in the very tail end of the distribution with only 5.0 × 10^−4^ iterations resulting in a coefficient greater than or equal to that from the original linear regression (0.1426). Thus, significance based on the randomization tests was concordant with the p-values from the regression analysis.

### Pre- and post-copulatory mating isolation

Males courted conspecific females more frequently than heterotypic or heterospecific females; however, these differences were only significant for *D. m. sonorensis* males (Additional file 2: Table S5a). Courtship acceptance by females followed a similar pattern to male courtship frequencies for *D. m. baja*, *D. m. wrigleyi*, and *D. arizonae* (Additional file 2: Table S6a), but not for *D. m. mojavensis* and *D. m. sonorensis*. Female *D. m. mojavensis* accepted a higher frequency of courtship attempts when mated to heterotypic males than conspecific males, but this difference was not significant. *Drosophila mojavensis sonorensis* females accepted courtship attempts from conspecific and heterotypic males at similar frequencies, but the frequency dropped when mated with *D. arizonae* males. For brevity and clarity, data for male courtship frequencies, female acceptance frequencies, and mean number of offspring are provided in Additional file 2: Tables S5-7.

## Discussion

I conducted reciprocal mate trials among taxa in the *D. mojavensis* species cluster to test the hypothesis that lineage specific size and shape differences of the male aedeagus play a role in mate recognition and successful copulatory interactions. I found that when genetic distance, shape difference, and size difference were considered together, the frequency of pseudocopulation was significantly correlated with aedeagus size, with a minimal role for shape. Further, comparison of pseudocopulation frequencies among taxa revealed that the direction of the size difference was important. In heterotypic and heterospecific crosses, pseudocopulation was more frequent when the mating interaction involved males that had a larger aedeagus, on average, than males of the females’ own species. Alternatively, in copulatory interactions where the male aedeagus was smaller, on average, than those of the females’ own species, the mating interactions began normally but often ended with the male and female unable to disengage from the mating position.

### Selection for the right fit

Based on patterns of aedeagus shape variation in the *D. mojavensis* species cluster, Richmond et al. hypothesized that aedeagus shape was involved in mate recognition [21]. If aedeagus form is important for mate recognition, then I would expect that lineage specific differences of the aedeagus to result in failed copulatory interactions in cross-taxon matings. In the current paper, I investigated two aspects of aedeagus form, size and shape, and tested whether differences associated with these variables were correlated with failed mating interactions, or pseudocopulation. After controlling for genetic relatedness, size was the only factor that had a significant effect on the frequency of pseudocopulation. This finding was further supported by analyses comparing pseudocopulation frequencies among taxa and mate groups.

In heterospecific crosses between *D. mojavensis* females and *D. arizonae* males, the frequency of Type I pseudocopulation was significantly different from conspecific and heterotypic crosses (Table 3), and approached 100% for all subspecies (except *D. m. mojavensis* where it was 75%). Because the aedeagus of *D. arizonae* is bigger and more robust than in any of the *D. mojavensis* subspecies (Figure 1), these data support the finding that Type I pseudocopulation is due to a size incompatibility. A similar situation was seen in *Parafontaria* millipedes, where copulatory interactions involving heterospecific males with larger gonopods (male intromittent structure) were not successful [22]. However, in the millipedes the copulatory interaction could not occur because the gonopod was too large to fit into the opening of the female reproductive system. In the crosses with *D. mojavensis* and *D. arizonae* the copulatory interaction was able to proceed past the point where the male aedeagus was inserted into the female but ended shortly thereafter.

The frequency of pseudocopulation was lower when *D. arizonae* females were paired with *D. mojavensis* subspecies males. In fact, the frequency of pseudocopulation was not significantly different when female *D. arizonae* were mated to conspecifics versus heterospecifics. There were several mate trials, however, where the copulatory interaction started successfully and mating proceeded as normal, but the male was unable to disengage his aedeagus at the end of the interaction. Unlike in the reciprocal cross where *D. arizonae* males with a larger aedeagus could not establish the appropriate copulatory position, *D. mojavensis* subspecies males with the smaller aedeagus could initiate copulation, which appeared to progress normally, but the copulatory interaction could not be terminated. Failed disengagement is suggestive of a mechanical incompatibility, and could result in trauma to the male and/or female reproductive system [23]. While these females were able to lay eggs, a more detailed investigation of the male and female reproductive organs following copulation is required to determine whether more localized trauma occurred.

In addition to investigating heterospecific copulatory interactions, I also conducted heterotypic crosses among *D. mojavensis* subspecies. Crosses involving *D. m. sonorensis* had higher frequencies of pseudocopulation than the other three subspecies. Specifically, pseudocopulation in *D. m. sonorensis* females was higher than all other heterotypic crosses when grouped by female taxon (Table 3). Based on the results presented here, it appears *D. m. sonorensis* females discriminate among males based on cues of aedeagus size. This finding would help explain why Richmond et al. [20] did not find evidence for a mate recognition role of aedeagus shape in *D. m. sonorensis*. If mate discrimination in *D. m. sonorensis* is based on size rather than shape, then patterns of aedeagus shape variation would not be concordant with those expected if aedeagus shape played a role in mate recognition.

While size is the predominant factor influencing successful mating interactions in the *D. mojavensis* species cluster, the results of the multiple linear regression, in combination with patterns of shape variation [20], suggest a potential role for aedeagus shape in heterotypic crosses. In *D. m. mojavensis* and *D. m. wrigleyi*, aedeagus size overlaps that of *D. m. sonorenesis* (Figure 3), suggesting that shape may contribute to Type I pseudocopulation in these crosses. Comparisons of aedeagus shape in these taxa revealed that differences between *D. m. sonorensis* and *D. m. mojavensis* were concentrated to the angle of the head and length of the nose of the aedeagus (Figure 1c), while *D. m. wrigleyi* differed primarily in the length and width of the nose (pairwise comparisons in figure six of [20]).

The occurrence of Type II pseudocopulation resulted from a series of failed mating attempts that eventually lead to a successful copulation. When a failed mating attempt occurred the male would either fall to the bottom of the vial or he would begin courting and make a subsequent attempt right away. Thus, Type II pseudocopulation was scored as a byproduct of the rapid succession of events that could occur in a lab-based mate trial. When the two types of pseudocopulation were analyzed together the results of the multiple linear regression were similar, and both supported a significant effect of aedeagus size.

The evolution of aedeagus morphology in an ecologically similar group of cactophilic *Drosophila* in South America shows some similarity to aedeagus evolution in the *D. mojavensis* species cluster. Reproductive isolation between the two sister species, *D. buzzatii* and *D. koepferae*, is incomplete (F1 hybrids can be produced in the lab), and analysis of aedeagus variation shows discrete size and shape differences [24]. In addition, both species show enhanced differences where they are sympatric [25], which suggests that the aedeagus is involved in mate recognition. A detailed study of aedeagus variation in *D. buzzatii* throughout its distribution in Argentina, however, suggested continuous directional selection consistent with sexual selection hypotheses rather than mate recognition [26]. An alternative explanation is that *D. buzzatii* populations are in the process of diverging and are thus experiencing a punctuated bout of directional selection, as would be predicted by mate recognition, that will be followed by a period of stabilizing selection. Further analysis of the mating interaction and pseudocopulation frequencies within and among *D. koepferae* and *D. buzzatii* would provide a comparative analysis of aedeagus evolution and provide insight into its role in mate recognition in incipient species.

The potential roles of other copulatory courtship interactions such as cuticular hydrocarbons, male grasping and stroking behaviors, and male courtship songs also should be considered. Variation in cuticular hydrocarbon profiles among the *D. mojavensis* subspecies has been documented and is suggested to play a role in sexual isolation [27-29]; but these cues serve a more critical role as pre-copulatory isolating mechanism [30]. Likewise, male courtship in many *Drosophila* species consists of a series of male stroking and grasping behaviors while the male is orienting himself behind the female, which, typically occurs before the male mounts the female [31]. Certainly successful mating interactions depend on a variety of cues, but the specific aspect of copulation being investigated here occurs after the above pre-copulatory isolating variables have been overcome.

The frequency of Type I pseudocopulation in conspecific crosses was generally lower than in heterotypic crosses for all subspecies except *D. m. baja*. One possible explanation is the recent range expansion of *D. arizonae* into Baja California Sur. Collection of low numbers of *D. arizonae* at the southern tip of the Baja Peninsula was first reported by Markow [31] and Heed [32]. However, recent collections at these same localities by the author and by T. A. Markow (2012-2013) reflect a much higher abundance of *D. arizonae*. A recent occurrence of novel sympatry of *D. m. baja* and *D. arizonae* has the potential to alter the mating dynamics of these species in this region with increased selection on pre- and post-copulatory isolating mechanisms. Additional work in my laboratory is taking advantage of this opportunity and preliminary experiments are currently underway.

Investigating the dynamics of heterotypic crosses in the *D. mojavensis* species cluster is particularly useful because all data collected to date suggests these lineages are incipient species. The current study establishes that the primary factor resulting in failed mating interactions in this group is aedeagus size, with a secondary role for aedeagus shape. These results show that morphological evolution of the aedeagus of *D. arizonae* and among *D. mojavensis* subspecies could contribute to reproductive isolation as a result of failed copulatory interactions.

## Conclusions

Our understanding of how genitalia evolve and their role in reproductive isolation and speciation remains incomplete. Taxonomic groups such as the *D. mojavensis* species cluster provide a unique opportunity to analyze morphological evolution at multiple levels of divergence. These types of studies coupled with additional analyses using scanning electron microscopy [33] and micro-CT of genitalia interactions [34], laser ablation techniques [35], and analysis of the innervation of male and female reproductive organs [36] will continue to improve our understanding of the mating interaction and its associated morphology.

## Methods

### Pseudocopulation rates

I used laboratory strains of each of the four *D. mojavensis* subspecies and one strain of *D. arizonae* (Additional file 2: Table S1). Virgin flies were obtained by initiating controlled-density cultures in half-pint bottles with 10 male-female pairs. After four days the adults were removed. Virgin males and females were collected within 20 hours of eclosion and aged for 9 days in yeasted vials, with no more than 10 flies per vial. All laboratory rearing was performed on banana medium at 23°C with a 12 hour light:dark cycle. Mate trials were started at 9:00 am (15 minutes after the beginning of the light cycle) at 23°C. At least 20 mate trials were conducted for all possible reciprocal pairwise combinations within the four *D. mojavensis* subspecies and with *D. arizonae.* At least three same (sub)species mate trials were conducted on each day of mating trials. If three conspecific trials resulted in no successful matings then data from the entire day would be excluded; however, this scenario did not occur during the study.

Single females were allowed to acclimate to their test vial for 24 hours before a single male was introduced by aspiration. For each mate trial between a single male and a single female, males were allowed three copulatory attempts. If all three resulted in unsuccessful copulation the trial was scored as Type I pseudocopulation. If a successful mating was preceded by at least one unsuccessful mounting attempt it was scored as Type II pseudocopulation. The frequency of Type I and II pseudocopulation was calculated using only those mate trials where the female accepted a male’s courtship; thus, these frequencies only represent unsuccessful copulation attempts, and do not include pre-copulatory mating propensity. In addition to pseudocopulation behavior, I also recorded the following pre-copulatory variables: time to courtship initiation, first copulatory attempt (prompted by a stereotypic female wing-spreading invitation), as well as the number of offspring resulting from all copulation types. Four days after mate trials were completed; females were transferred to new vials supplemented with yeast. Pre- and post-copulatory variables were included in data collection to provide an inclusive picture of reproductive isolation mechanisms that potentially occur in conjunction with pseudocopulation. For brevity, these data are reported in Additional file 2. The research presented here was conducted on laboratory cultures of *Drosophila* species and does not require ethics approval.

### Morphological and genetic distance

Aedeagus shape differences between taxonomic groups were quantified by calculating Mahalanobis distances between taxa using the principal component scores from the seven effective principal components from outline shape analysis in Richmond *et al.* [20]. Size difference was calculated using the average size for each taxon reported in Richmond et al. [20]. Size differences were calculated relative to the female in the cross, i.e. if the cross-taxon male had a larger aedeagus than males of her own species the difference was positive. If the cross-taxon male had a smaller aedeagus than males of her own species the difference was negative.

Genetic distance between taxa was determined by generating a species tree using a subset of the data from Machado et al. [11]. The four nuclear markers used allowed for the largest inclusion of individuals (least missing data): 996, 5307, A4125, and X100. Models of molecular evolution for each marker were determined using jModelTest v0.1.1 [37] and implemented in BEAST v1.6.2 [38] using the *BEAST option [39]. Genetic distances between taxa were based on the branch lengths of the resulting species tree. Topology of the species tree was identical to that presented in Machado et al. [11] (Additional file 2: Figure S1).

Two multiple linear regression analyses were done with size and shape distance of the aedeagus, and genetic distance as independent variables. In the first analysis the frequency of Type I pseudocopulation was the dependent variable. The second analysis included the frequency of both Type I and Type II pseudocopulation added together as a single dependent variable. All data were analyzed in R [40]. Pseudocopulation rates were compared among taxa within mate groups (conspecific/contypic, heterotypic, and heterospecific; see definitions in Table 4), and among mate groups within each taxon using Fisher’s exact test. I used the fmsb v0.4.1 package (Functions for medical statistics book with some demographic data) [41] for the Fisher exact tests, and the HDMD package v1.2 (High dimension molecular data) [42] to calculate Mahalanobis distances representing aedeagus shape differences.

**Table 4:** **Definition of terms used to describe mate trial groupings**

**Comparison among:**	**Definition**
Mating Groups	Three types: conspecific (includes contypic in the case of *D. mojavensis* subspecies), heterotypic, and heterospecific.
Cross Types	25 different types: all reciprocal pairwise comparisons, e.g. *D. m. mojavensis* x *D. m. baja*. Female in cross is always listed first.
Taxa	Five designations: *D. arizone*, *D. m. baja*, *D. m. mojavensis*, *D. m. sonorensis*, and *D. m. wrigleyi*. When grouped by female taxon, includes all mate trials with a female from specified taxon. Same when grouped by male taxon.

## Availability of supporting data

The data sets supporting the results of this article are available in the Dryad Digital Repository, doi:10.5061/dryad.4jg3b [43].

## Additional files

Additional file 1: Video S1. Type I pseudocopulation. Additional file 2:
**Tables S1-S8 and Figure S1.**
Additional file 3: Video S2. Disengagement difficulties between male and female.
