# Supplementary material for: The role of aedeagus size and shape in failed mating interactions among recently diverged taxa in the Drosophila mojavensis species cluster
Source: BMC Evol Biol. 2014 Dec 10;14:255. doi: 10.1186/s12862-014-0255-3 (PMC4269899; doi:10.1186/s12862-014-0255-3)
Supplement: Additional file 2: — Tables S1-S8 and Figure S1. [file 12862_2014_255_MOESM2_ESM.docx]

**Additional File 2: Supplementary Data**

**Table S1.** Stock numbers and locality data for flies used in the study. (Note: there was a mistake in Richmond et al. (2012) for the *D. arizonae* stock used. That paper listed 15081-1271.18 but it should have been 15081-1271.21, the same stock used for the current study).

| **Species** | **DSSC Stock Number** | **Locality (year collected)** |
| --- | --- | --- |
| *D. arizonae* | 15081-1271.18 | Tucson, AZ, USA (2004) |
| *D. m. baja* | 15081-1351.30 | Punta Prieta, Baja California, Mexico (2008) |
| *D. m. mojavensis* | 15081-1352.01 | Anza Borrego Desert, CA, USA |
| *D. m. sonorensis* | 15081-1352.26 | Agiabampo Bay, Sonora, Mexico (2003) |
| *D. m. wrigleyi* | 15081-1352.02 | Catalina Island, CA, USA (1991) |

**Table S2.** Sample sizes: number of total mate trials, and (/) number of trials where male courted, female accepted, and male made mating attempt (= denominator when calculating normal copulation, Pseudocopulation Type I, and Pseudocopulatoin Type II frequencies). Mate groups depicted in different colored regions (white = conspecific, light gray = heterotypic, dark gray = heterospecific).

|  | **Male** | *D. m. baja* | *D. m. mojavensis* | *D. m. sonorensis* | *D. m. wrigleyi* | *D. arizonae* |
| --- | --- | --- | --- | --- | --- | --- |
| **Female** |  |  |  |  |  |  |
| *D. m. baja* |  | 20/20 | 22/20 | 20/18 | 20/18 | 20/19 |
| *D. m. mojavensis* |  | 22/17 | 40/36 | 23/19 | 23/23 | 20/16 |
| *D. m. sonorensis* |  | 23/16 | 20/16 | 23/19 | 20/17 | 20/4 |
| *D. m. wrigleyi* |  | 20/17 | 31/27 | 20/20 | 27/27 | 20/9 |
| *D. arizonae* |  | 20/11 | 20/15 | 20/0 | 20/17 | 23/20 |

**Table S3a.** Frequency of normal copulation by male species/subspecies listed as a percentage. Symbols as in Supp. Fig. 2a. (N/A denotes no possibility of normal copulation for *D. arizonae* x *D. m. sonorensis* crosses).

| **Species** | **%CS** | **%HT** | **%HS^** |
| --- | --- | --- | --- |
| *D. m. baja* | 60.0 | 52.0 | 63.6 |
| *D. m. mojavensis** | 85.7 | 57.1 | 93.3 |
| *D. m. sonorensis* | 68.4 | 67.2 | N/A |
| *D. m. wrigleyi** | 77.8 | 60.3 | 29.4 |
| *D. arizonae** | 90.0 | - | 0.0 |

**Table S3b.** Frequency of normal copulation by female species/subspecies listed as a percentage. Symbols as in Supp. Fig. 2a.

| **Species** | **%CS** | **%HT^** | **%HS^** |
| --- | --- | --- | --- |
| *D. m. baja** | 60.0 | 69.6 | 0.0 |
| *D. m. mojavensis** | 85.7 | 65.0 | 0.0 |
| *D. m. sonorensis* | 68.4 | 38.8 | 0.0 |
| *D. m. wrigleyi** | 77.8 | 61.0 | 0.0 |
| *D. arizonae* | 90.0 | - | 60.5 |

**Table S3c.** Frequency of normal copulation by cross, listed as a percentage. Regions colored as in Supp. Table 2. (N/A denotes a denominator of zero for *D. arizonae* x *D. m. sonorensis* crosses).

|  | **Male** | *D. m. baja* | *D. m. mojavensis* | *D. m. sonorensis* | *D. m. wrigleyi* | *D. arizonae* |
| --- | --- | --- | --- | --- | --- | --- |
| **Female** |  |  |  |  |  |  |
| *D. m. baja* |  | 60.0 | 55.0 | 88.9 | 66.7 | 0.0 |
| *D. m. mojavensis* |  | 70.6 | 85.7 | 55.0 | 69.6 | 0.0 |
| *D. m. sonorensis* |  | 31.2 | 43.7 | 68.5 | 41.2 | 0.0 |
| *D. m. wrigleyi* |  | 53.0 | 66.7 | 60.0 | 77.8 | 0.0 |
| *D. arizonae* |  | 63.6 | 93.3 | N/A | 29.4 | 90.0 |

**Table S4.** Frequency of pseudocopulation Type II by cross, listed as percentages. Regions colored as in Supp. Table 2.

|  | **Male** | *D. m. baja* | *D. m. mojavensis* | *D. m. sonorensis* | *D. m. wrigleyi* | *D. arizonae* |
| --- | --- | --- | --- | --- | --- | --- |
| **Female** |  |  |  |  |  |  |
| *D. m. baja* |  | 10.0 | 45.0 | 5.5 | 33.3 | 0.0 |
| *D. m. mojavensis* |  | 5.9 | 14.3 | 10.0 | 13.0 | 25.0 |
| *D. m. sonorensis* |  | 18.8 | 25.0 | 21.0 | 23.5 | 0.0 |
| *D. m. wrigleyi* |  | 35.2 | 25.9 | 30.0 | 14.8 | 0.0 |
| *D. arizonae* |  | 18.2 | 6.7 | N/A | 41.2 | 10.0 |

**Table S5a.** Male courtship frequencies by male species/subspecies listed as a percentage (CS = conspecific pairing, HT = heterotypic pairing HS = heterospecific pairing). * = significant among mating group type (horizontal), and ^ = significant among taxa (vertical) using Fisher’s Exact test for pairwise comparisons with Bonferroni correction (α = 0.05). (“-“ denotes no HT cross exists for *D. arizonae.*)

| **Species** | **%CS** | **%HT** | **%HS** |
| --- | --- | --- | --- |
| *D. m. baja* | 100.0 | 84.6 | 80.0 |
| *D. m. mojavensis* | 97.5 | 97.3 | 90.0 |
| *D. m. sonorensis** | 100.0 | 98.4 | 75.0 |
| *D. m. wrigleyi* | 100.0 | 93.7 | 100.0 |
| *D. arizonae* | 95.7 | - | 92.5 |

**Table S5b.** Male courtship attempt frequencies by cross listed as a percentage. Regions colored as in Supp. Table 2.

|  | **Male** | *D. m. baja* | *D. m. mojavensis* | *D. m. sonorensis* | *D. m. wrigleyi* | *D. arizonae* |
| --- | --- | --- | --- | --- | --- | --- |
| **Female** |  |  |  |  |  |  |
| *D. m. baja* |  | 100.0 | 100.0 | 100.0 | 90.0 | 100.0 |
| *D. m. mojavensis* |  | 81.8 | 97.5 | 95.2 | 100.0 | 95.0 |
| *D. m. sonorensis* |  | 78.3 | 95.0 | 100.0 | 90.0 | 90.0 |
| *D. m. wrigleyi* |  | 95.0 | 96.8 | 100.0 | 100.0 | 85.0 |
| *D. arizonae* |  | 80.0 | 90.0 | 75.0 | 100.0 | 95.7 |

**Table S6a.** Female courtship acceptance frequencies by female species/subspecies listed as a percentage. Symbols as in Supp. Fig. 2a.

| **Species** | **%CS** | **%HT** | **%HS^** |
| --- | --- | --- | --- |
| *D. m. baja* | 100.0 | 93.3 | 95.0 |
| *D. m. mojavensis* | 89.7 | 98.4 | 84.2 |
| *D. m. sonorensis** | 82.6 | 89.1 | 22.2 |
| *D. m. wrigleyi** | 100.0 | 92.8 | 52.9 |
| *D. arizonae** | 90.9 | - | 62.3 |

**Table S6b.** Female courtship acceptance frequencies by cross listed as a percentage. Regions colored as in Supp. Table 2.

|  | **Male** | *D. m. baja* | *D. m. mojavensis* | *D. m. sonorensis* | *D. m. wrigleyi* | *D. arizonae* |
| --- | --- | --- | --- | --- | --- | --- |
| **Female** |  |  |  |  |  |  |
| *D. m. baja* |  | 100.0 | 90.9 | 90.0 | 100.0 | 95.0 |
| *D. m. mojavensis* |  | 94.4 | 89.7 | 100.0 | 100.0 | 84.2 |
| *D. m. sonorensis* |  | 88.9 | 84.2 | 82.6 | 94.4 | 22.2 |
| *D. m. wrigleyi* |  | 89.5 | 90.0 | 100.0 | 100.0 | 52.9 |
| *D. arizonae* |  | 68.8 | 83.3 | 0.0 | 85.0 | 90.9 |

**Table S7a.** Mean number of offspring (and associated standard error) resulting from successful matings by female taxon.

| **Taxon** | **CS** | **HT** |
| --- | --- | --- |
| *D. m. baja* | 25.0 (7.9) | 30.07 (3.6) |
| *D. m. mojavensis* | 59.8 (4.1) | 51.00 (3.4) |
| *D. m. sonorensis* | 24.9 (6.8) | 44.57 (4.3) |
| *D. m. wrigleyi* | 24.4 (5.1) | 30.37 (3.2) |
| *D. arizonae* | 36.8 (7.5) | - |

**Table S7b.** Mean number of offspring (and associated standard error) resulting from successful mating by mate cross. Regions colored as in Supp. Table 2.

|  | **Male** | *D. m. baja* | *D. m. mojavensis* | *D. m. sonorensis* | *D. m. wrigleyi* | *D. arizonae* |
| --- | --- | --- | --- | --- | --- | --- |
| **Female** |  |  |  |  |  |  |
| *D. m. baja* |  | 25.0 (7.9) | 30.7 (5.2) | 35.1 (6.1) | 25.8 (5.2) | N/A |
| *D. m. mojavensis* |  | 33.1 (5.8) | 59.8 (4.1) | 44.7 (5.6) | 65.1 (4.4) | 9.0 (8.8) |
| *D. m. sonorensis* |  | 36.1 (7.6) | 58.9 (6.1) | 24.9 (6.8) | 34.7 (6.4) | N/A |
| *D. m. wrigleyi* |  | 17.6 (5.4) | 46.0 (4.4) | 0.0 | 24.4 (5.1) | N/A |
| *D. arizonae* |  | 0.0 | 0.0 | N/A | 0.0 | 36.8 (7.5) |

**Table S8.** Genetic distance calculated from species tree branch lengths (upper diagonal), and Mahalanobis distance for aedeagus shape calculated using principal components (lower diagonal). See text for more detail on the data used to calculate distances. Regions colored as in Supp. Table 2.

|  | **Male** | *D. m. baja* | *D. m. mojavensis* | *D. m. sonorensis* | *D. m. wrigleyi* | *D. arizonae* |
| --- | --- | --- | --- | --- | --- | --- |
| **Female** |  |  |  |  |  |  |
| *D. m. baja* |  |  | 0.00613 | 0.00087 | 0.00613 | 0.01993 |
| *D. m. mojavensis* |  | 0.006333 |  | 0.00613 | 0.00153 | 0.01994 |
| *D. m. sonorensis* |  | 0.001506 | 0.003059 |  | 0.00613 | 0.01993 |
| *D. m. wrigleyi* |  | 0.008176 | 0.002977 | 0.003960 |  | 0.01994 |
| *D. arizonae* |  | 0.085585 | 0.059511 | 0.073799 | 0.054569 |  |

**Figure S1.** Species tree with posterior probabilities estimated in *BEAST using a subset of the multilocus data from Machado et al. (2006).
